# (2*E*,3*E*)-3-(Pyrazin-2-yloxyimino)butan-2-one oxime

**DOI:** 10.1107/S1600536808027128

**Published:** 2008-08-30

**Authors:** Ju Na Chen, Lin Yan Yang

**Affiliations:** a Naval Aeronautical and Astronautical University, Yantai 264001, People’s Republic of China; b Department of Chemistry, Shandong Normal University, Jinan 250014, People’s Republic of China

## Abstract

In the title compound, C_8_H_10_N_4_O_2_, all non-H atoms are nearly coplanar [maximum deviation 0.1256 (16) Å for the methyl C furthest from the ring]. Inter­molecular O—H⋯N hydrogen bonds link adjacent mol­ecules into a one-dimensional zigzag chain along the *c* axis. There is also a weak π–π stacking inter­action between neighbouring pyrazine rings, with a centroid–centroid distance of 4.0432 (15) Å.

## Related literature

For related papers, see: Wang *et al.* (2008 ▶); Khan *et al.* (1993 ▶).
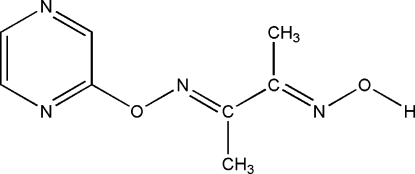


## Experimental

### 

#### Crystal data

C_8_H_10_N_4_O_2_
*M* *_r_* = 194.20
Monoclinic, 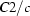

*a* = 18.174 (4) Å
*b* = 10.962 (3) Å
*c* = 13.271 (3) Å
β = 132.217 (3)°
*V* = 1958.1 (8) Å^3^
*Z* = 8
Mo *K*α radiation
μ = 0.10 mm^−1^
*T* = 298 (2) K
0.48 × 0.40 × 0.28 mm

#### Data collection

Bruker SMART APEX CCD diffractometer
Absorption correction: multi-scan (*SADABS*; Sheldrick, 1996 ▶) *T*_min_ = 0.954, *T*_max_ = 0.973
5536 measured reflections
2134 independent reflections
1431 reflections with *I* > 2σ(*I*)
*R*_int_ = 0.025

#### Refinement

*R*[*F* ^2^ > 2σ(*F* ^2^)] = 0.052
*wR*(*F* ^2^) = 0.172
*S* = 1.06
2134 reflections
129 parameters
H-atom parameters constrained
Δρ_max_ = 0.19 e Å^−3^
Δρ_min_ = −0.16 e Å^−3^

### 

Data collection: *SMART* (Bruker, 1997 ▶); cell refinement: *SAINT* (Bruker, 1997 ▶); data reduction: *SAINT*; program(s) used to solve structure: *SHELXTL* (Sheldrick, 2008 ▶); program(s) used to refine structure: *SHELXTL*; molecular graphics: *SHELXTL*; software used to prepare material for publication: *SHELXTL* and local programs.

## Supplementary Material

Crystal structure: contains datablocks I, global. DOI: 10.1107/S1600536808027128/at2614sup1.cif

Structure factors: contains datablocks I. DOI: 10.1107/S1600536808027128/at2614Isup2.hkl

Additional supplementary materials:  crystallographic information; 3D view; checkCIF report

## Figures and Tables

**Table 1:** Hydrogen-bond geometry (Å, °)

*D*—H⋯*A*	*D*—H	H⋯*A*	*D*⋯*A*	*D*—H⋯*A*
O1—H1⋯N2^i^	0.81	1.98	2.774 (2)	166

Symmetry code: (i) 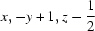.

**Fig. 1.**
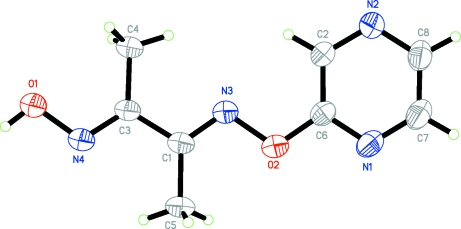
Structure of title compound with the atom-numbering scheme. Displacement ellipsoids are drawn at the 30% probability level.

**Fig. 2.**
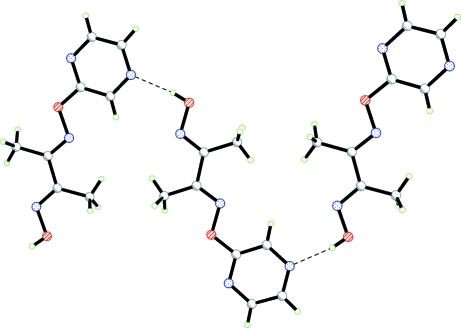
Hydrogen bonds (dashed lines) between neighbouring molecules.

## supplementary crystallographic information

### Comment

Pyrazine, (2E,3E)-butane-2,3-dione dioxime and their derivatives belong to useful compounds and a large number of complexes have been synthesized with them as ligands (Wang *et al.*, (2008) and Khan *et al.* (1993)). We are interested in complexes with the title compound as ligand, therefore we synthesized the title compound and obtained its crystal structure (I).

Fig. 1 shows the molecular structure of the title compound and the all of non-hydrogen atoms define a plane with a maximum deviation of 0.1256 (16) Å for atom C4. There is a weak π-π stacking interaction involving symmetry-related pyrazine rings, which resulted in the formation of a dimer of two neighbor molecules, and the relevant distances being Cg1···Cg1^i^ = 4.0432 (15) Å and Cg1···Cg1^i^_perp_ = 3.248 Å and α = 5.71°; [symmetry code: (i) -x, y, 1/2-z; Cg1 is the centroid of the N1N2/C2C6C7C8 ring, Cg1···Cg2^i^_perp_ is the perpendicular distance from ring Cg1 to ring Cg1^i^; α is the dihedral angle between ring plane Cg1 and ring plane Cg1^i^]. In addition to the π-π interaction there exists O1—H1···N2^ii^ [symmetry code: (ii) *x*, 1-*y*,-1/2+*z*] hydrogen bond and it give rise a one-dimensional zigzag chain along c axis as shown in Fig. 2 (Table 1).

### Experimental

Powder (2E,3E)-butane-2,3-dione O^3^-(2-pyrazyl) dioxime (0.3720 g, 1.92 mmole) was dissolved in 20 ml solution containing 10 ml chloroform and 10 ml me thanol, and the colorless single crystals were obtained after the solution had been allowed to stand at room temperature for a month.

### Refinement

Oxygen-bound H atom was located in a difference Fourier map, and refined as riding in its as found position with O—H = 0.81 Å, *U*_iso_(H) = 1.5*U*_eq_(O). Other H atoms were placed in calculated positions (C—H = 0.96 Å for methyl group and C—H = 0.93 Å for pyrazinyl H atoms) and refined as riding with *U*_iso_ = 1.5*U*_eq_(C) for methyl H and *U*_iso_ = 1.2*U*_eq_(C) for pyrazinyl H atoms.

### Crystal data

**Table:**

C_8_H_10_N_4_O_2_	*F*_000_ = 816
*M**_r_* = 194.20	*D*_x_ = 1.318 Mg m^−^^3^
Monoclinic, *C*2/*c*	Mo *K*α radiation λ = 0.71073 Å
Hall symbol: -C 2yc	Cell parameters from 1722 reflections
*a* = 18.174 (4) Å	θ = 2.4–25.5º
*b* = 10.962 (3) Å	µ = 0.10 mm^−^^1^
*c* = 13.271 (3) Å	*T* = 298 (2) K
β = 132.217 (3)º	Block, colourless
*V* = 1958.1 (8) Å^3^	0.48 × 0.40 × 0.28 mm
*Z* = 8	

### Data collection

**Table:**

Bruker SMART APEX CCD diffractometer	2134 independent reflections
Radiation source: fine-focus sealed tube	1431 reflections with *I* > 2σ(*I*)
Monochromator: graphite	*R*_int_ = 0.025
*T* = 293(2) K	θ_max_ = 27.0º
φ and ω scans	θ_min_ = 2.4º
Absorption correction: multi-scan(SADABS; Sheldrick, 1996)	*h* = −22→23
*T*_min_ = 0.954, *T*_max_ = 0.973	*k* = −13→13
5536 measured reflections	*l* = −16→9

### Refinement

**Table:**

Refinement on *F*^2^	Secondary atom site location: difference Fourier map
Least-squares matrix: full	Hydrogen site location: inferred from neighbouring sites
*R*[*F*^2^ > 2σ(*F*^2^)] = 0.052	H-atom parameters constrained
*wR*(*F*^2^) = 0.172	*w* = 1/[σ^2^(*F*_o_^2^) + (0.1*P*)^2^ + 0.0347*P*] where *P* = (*F*_o_^2^ + 2*F*_c_^2^)/3
*S* = 1.06	(Δ/σ)_max_ = 0.045
2134 reflections	Δρ_max_ = 0.19 e Å^−^^3^
129 parameters	Δρ_min_ = −0.16 e Å^−^^3^
Primary atom site location: structure-invariant direct methods	Extinction correction: none

### Special details

**Table:**

Geometry. All e.s.d.'s (except the e.s.d. in the dihedral angle between two l.s. planes) are estimated using the full covariance matrix. The cell e.s.d.'s are taken into account individually in the estimation of e.s.d.'s in distances, angles and torsion angles; correlations between e.s.d.'s in cell parameters are only used when they are defined by crystal symmetry. An approximate (isotropic) treatment of cell e.s.d.'s is used for estimating e.s.d.'s involving l.s. planes.
Refinement. Refinement of *F*^2^ against ALL reflections. The weighted *R*-factor *wR* and goodness of fit *S* are based on *F*^2^, conventional *R*-factors *R* are based on *F*, with *F* set to zero for negative *F*^2^. The threshold expression of *F*^2^ > σ(*F*^2^) is used only for calculating *R*-factors(gt) *etc*. and is not relevant to the choice of reflections for refinement. *R*-factors based on *F*^2^ are statistically about twice as large as those based on *F*, and *R*- factors based on ALL data will be even larger.

### Fractional atomic coordinates and isotropic or equivalent isotropic displacement parameters (Å^2^)

**Table:**

	*x*	*y*	*z*	*U*_iso_*/*U*_eq_	
C1	0.12820 (11)	0.43770 (16)	0.26471 (15)	0.0584 (4)	
C2	0.12053 (12)	0.20436 (15)	0.48282 (18)	0.0630 (5)	
H2	0.1234	0.2871	0.5006	0.076*	
C3	0.12962 (12)	0.56843 (16)	0.29319 (16)	0.0583 (4)	
C4	0.13696 (16)	0.60601 (15)	0.4082 (2)	0.0753 (6)	
H4A	0.1945	0.6566	0.4702	0.113*	
H4B	0.1426	0.5347	0.4550	0.113*	
H4C	0.0785	0.6508	0.3730	0.113*	
C5	0.12486 (18)	0.39842 (18)	0.1538 (2)	0.0813 (6)	
H5A	0.1707	0.3325	0.1857	0.122*	
H5B	0.1428	0.4658	0.1278	0.122*	
H5C	0.0589	0.3718	0.0766	0.122*	
C6	0.12287 (11)	0.16599 (15)	0.38501 (16)	0.0584 (4)	
C7	0.11298 (15)	−0.02865 (17)	0.4241 (2)	0.0813 (6)	
H7	0.1096	−0.1113	0.4056	0.098*	
C8	0.11035 (14)	0.00630 (17)	0.5209 (2)	0.0764 (6)	
H8	0.1058	−0.0530	0.5666	0.092*	
N1	0.12018 (11)	0.05109 (14)	0.35580 (16)	0.0742 (5)	
N2	0.11418 (11)	0.12313 (13)	0.55072 (16)	0.0716 (5)	
N3	0.12995 (10)	0.36663 (12)	0.34221 (14)	0.0600 (4)	
N4	0.12369 (11)	0.64309 (14)	0.21366 (14)	0.0661 (4)	
O1	0.12332 (10)	0.76336 (11)	0.24571 (14)	0.0821 (4)	
H1	0.1130	0.8035	0.1863	0.123*	
O2	0.12790 (9)	0.24281 (11)	0.30873 (12)	0.0687 (4)	

### Atomic displacement parameters (Å^2^)

**Table:**

	*U*^11^	*U*^22^	*U*^33^	*U*^12^	*U*^13^	*U*^23^
C1	0.0597 (9)	0.0721 (11)	0.0488 (9)	0.0017 (7)	0.0386 (8)	−0.0016 (8)
C2	0.0751 (11)	0.0516 (9)	0.0704 (10)	0.0022 (7)	0.0522 (9)	−0.0027 (8)
C3	0.0666 (10)	0.0658 (11)	0.0556 (9)	0.0004 (7)	0.0464 (8)	−0.0025 (8)
C4	0.1091 (14)	0.0730 (13)	0.0787 (12)	−0.0015 (10)	0.0774 (12)	−0.0034 (9)
C5	0.1160 (16)	0.0838 (14)	0.0661 (11)	0.0031 (10)	0.0701 (12)	−0.0048 (9)
C6	0.0540 (9)	0.0571 (10)	0.0575 (9)	−0.0021 (7)	0.0348 (8)	−0.0077 (8)
C7	0.0914 (13)	0.0554 (11)	0.0976 (14)	−0.0045 (9)	0.0637 (12)	−0.0121 (11)
C8	0.0828 (12)	0.0547 (12)	0.0963 (15)	−0.0014 (8)	0.0620 (12)	0.0023 (10)
N1	0.0824 (10)	0.0619 (9)	0.0805 (10)	−0.0055 (7)	0.0556 (9)	−0.0162 (8)
N2	0.0881 (10)	0.0589 (10)	0.0842 (11)	0.0021 (7)	0.0647 (10)	0.0014 (7)
N3	0.0698 (9)	0.0601 (9)	0.0577 (8)	−0.0018 (6)	0.0459 (8)	−0.0046 (7)
N4	0.0831 (10)	0.0687 (10)	0.0660 (9)	−0.0001 (7)	0.0581 (8)	−0.0013 (7)
O1	0.1258 (11)	0.0661 (9)	0.0898 (9)	−0.0013 (7)	0.0869 (9)	−0.0011 (7)
O2	0.0858 (8)	0.0665 (8)	0.0648 (8)	−0.0039 (6)	0.0550 (7)	−0.0097 (6)

### Geometric parameters (Å, °)

**Table:**

C1—N3	1.273 (2)	C5—H5B	0.9600
C1—C3	1.478 (3)	C5—H5C	0.9600
C1—C5	1.495 (2)	C6—N1	1.309 (2)
C2—N2	1.326 (2)	C6—O2	1.366 (2)
C2—C6	1.392 (2)	C7—N1	1.327 (2)
C2—H2	0.9300	C7—C8	1.372 (3)
C3—N4	1.282 (2)	C7—H7	0.9300
C3—C4	1.497 (2)	C8—N2	1.328 (2)
C4—H4A	0.9600	C8—H8	0.9300
C4—H4B	0.9600	N3—O2	1.4209 (18)
C4—H4C	0.9600	N4—O1	1.3868 (18)
C5—H5A	0.9600	O1—H1	0.8074
			
N3—C1—C3	113.57 (14)	C1—C5—H5C	109.5
N3—C1—C5	125.55 (17)	H5A—C5—H5C	109.5
C3—C1—C5	120.88 (15)	H5B—C5—H5C	109.5
N2—C2—C6	120.13 (17)	N1—C6—O2	112.42 (14)
N2—C2—H2	119.9	N1—C6—C2	123.25 (17)
C6—C2—H2	119.9	O2—C6—C2	124.33 (16)
N4—C3—C1	115.55 (14)	N1—C7—C8	122.47 (18)
N4—C3—C4	124.34 (16)	N1—C7—H7	118.8
C1—C3—C4	120.11 (14)	C8—C7—H7	118.8
C3—C4—H4A	109.5	N2—C8—C7	121.37 (18)
C3—C4—H4B	109.5	N2—C8—H8	119.3
H4A—C4—H4B	109.5	C7—C8—H8	119.3
C3—C4—H4C	109.5	C6—N1—C7	115.64 (16)
H4A—C4—H4C	109.5	C2—N2—C8	117.12 (17)
H4B—C4—H4C	109.5	C1—N3—O2	110.52 (14)
C1—C5—H5A	109.5	C3—N4—O1	111.69 (13)
C1—C5—H5B	109.5	N4—O1—H1	105.3
H5A—C5—H5B	109.5	C6—O2—N3	110.96 (12)
			
N3—C1—C3—N4	−177.17 (13)	C6—C2—N2—C8	0.0 (2)
C5—C1—C3—N4	2.7 (2)	C7—C8—N2—C2	0.1 (3)
N3—C1—C3—C4	2.5 (2)	C3—C1—N3—O2	179.69 (12)
C5—C1—C3—C4	−177.62 (17)	C5—C1—N3—O2	−0.2 (2)
N2—C2—C6—N1	−0.8 (2)	C1—C3—N4—O1	178.66 (13)
N2—C2—C6—O2	179.03 (15)	C4—C3—N4—O1	−1.0 (2)
N1—C7—C8—N2	0.5 (3)	N1—C6—O2—N3	179.56 (12)
O2—C6—N1—C7	−178.54 (14)	C2—C6—O2—N3	−0.3 (2)
C2—C6—N1—C7	1.3 (2)	C1—N3—O2—C6	−175.83 (12)
C8—C7—N1—C6	−1.2 (3)		

### Hydrogen-bond geometry (Å, °)

**Table:**

*D*—H···*A*	*D*—H	H···*A*	*D*···*A*	*D*—H···*A*
O1—H1···N2^i^	0.81	1.98	2.774 (2)	166

Symmetry codes: (i) *x*, −*y*+1, *z*−1/2.
